# Dental Professionals' Knowledge and Understanding of Intimate Partner Violence: A Pan‐Canadian Cross‐Sectional Survey

**DOI:** 10.1111/edt.70037

**Published:** 2025-11-28

**Authors:** Natalie Hamm, Liran Levin

**Affiliations:** ^1^ Bitonte College of Dentistry Northeast Ohio Medical University (NEOMED) Rootstown Ohio USA

**Keywords:** bruise, domestic abuse, head, neck, tooth injury

## Abstract

**Background:**

Intimate partner violence (IPV) is a global health concern, with over a quarter of women and girls worldwide experiencing IPV at least once in their lifetime. Previous research has indicated that dentists often lack the training and confidence to handle IPV‐related concerns in clinical settings. The purpose of this study was to examine dental professionals' knowledge, attitudes, and solutions offered to victims regarding IPV‐related concerns.

**Methods:**

A cross‐sectional study in a survey format was administered across Canada to collect responses from dentists, dental hygienists, dental therapists, and dental assistants. The questions in the survey were designed to evaluate professionals' knowledge of IPV, comfort levels addressing the topic with their patients, attitudes toward IPV, and knowledge of intervention resources.

**Results:**

Overall, 327 dental professionals completed the survey, with 183 dentists, 77 dental assistants, 60 dental hygienists, and 5 dental therapists. Most dental professionals indicated that they have not received training related to IPV at any point either during their education (88%) or career (81%). Furthermore, most dental professionals reported that they did not feel comfortable speaking to patients about IPV‐related concerns (51%) nor did they feel prepared to provide referrals or resources to victims. However, dental professionals generally feel that they do have a role in responding to IPV‐related events (55%) and would like more training on this topic (61%).

**Conclusions:**

Dental professionals largely believe that they are key responders to assist potential IPV victims and appear willing to take steps toward doing so. This study's findings also indicate that there is not currently widespread access to the tools, protocols, and educational resources that could facilitate this assistance.

## 1 Introduction

Intimate partner violence (IPV) is a global concern. Worldwide, approximately 6.2 million women and girls have reported violence within their intimate partner relationship [1] and 26% of women have been subjected to IPV from a male partner at least once in their life [2]. IPV is defined as behavior causing physical, sexual, or psychological harm within an intimate partner relationship [2]. Importantly, IPV includes violence perpetrated by both current and former partners [2]. Perpetrators often use acts of various forms of violence to gain power and control over their partner, such as physical, sexual, emotional or psychological, or financial harm [3]. All forms of violence used by perpetrators may present and potentially be identified in a dental setting.

Dental professionals and settings may play an important role in recognizing and helping victims of IPV [4]. Traumatic dental injuries, such as teeth and facial fractures, bruising and damage to the oral mucosa are commonly reported in victims of violent IPV [5]. Several studies have found injuries associated with IPV were more likely to occur in the head and face area, such as in the maxilla, zygomatic bone, and mandible, than in other areas of the body [6, 7]. One study found that a majority (38.7%) of injuries caused by domestic violence were in the head and neck region [8]. Another study examining radiology reports of 96 victims with facial injuries found that nasal fractures were the most commonly reported, followed by mandibular fractures, which made up 18.4% of facial fractures [9]. Research has shown significant correlations between domestic violence and tooth fractures as well [10]. This was also reflected in a recent report from social media perceptions of IPV [11].

The effects of IPV extend beyond injuries to the face and oral cavity and can impact the overall health of the oral cavity. Significant correlations between psychological abuse and poor oral health and periodontal status have been found [10]. Victims of IPV may also have poor oral health habits as a result of the abuse and its psychological effects. For example, victims of domestic violence were reported to perform less in terms of oral hygiene home care habits [10]. The lack of healthy oral behaviors may be a result of the stress and anxiety experienced by IPV victims and often might cause higher rates of dental caries, periodontal disease, and other oral conditions [10]. Increased stress and anxiety may also result in bruxing (tooth grinding) and temporo‐mandibular disorders (TMD) [12]. Research has shown that women exposed to IPV are 1.45 times more likely to develop TMD than women not exposed to IPV [13].

Despite clear connections between IPV and oral health, there is a lack of awareness and knowledge from dental professionals on the topic. For example, one study found that dentists' attitudes toward including IPV screening in their practice were negative [14]. Half of the dentists who participated reported that they could not provide a referral place for such patients. The same study also found that a low percentage of dentists included questions that screened for IPV through their health history form. Importantly, these results reflect an earlier study which found that 87% of dentists never screened for domestic violence in their patients [15]. Furthermore, in cases where dental professionals are willing to screen for IPV, they are often inhibited from doing so by significant barriers. Such barriers include the presence of a child or partner, fear of offending the patient, their own embarrassment, or time constraints [15, 16]. Other barriers may include resistance or stigma from dental professionals to include screening and aids in their practice [16]. Additionally, many dentists lack the necessary training to properly aid victims of IPV. One study reported that 53.2% of dentists received no training for detecting IPV and less than 4% of dentists reported more than 8 h of training, which was the maximum amount of training time recorded [14]. A lack of training time and education may prevent dental professionals from approaching and helping victims of IPV.

Current research is needed on dentists' and other dental professionals' abilities to recognize IPV and support such concerns (i.e., referral to shelters, police and/or legal knowledge). Many patients present to dentists with visible signs of abuse, but dentists lack screening and aids in their practice [16]. Thus, the aim of this study was to assess these gaps in order to focus future efforts to overcome existing barriers. Therefore, this study aimed to identify dental professionals' (1) knowledge about IPV, (2) willingness to screen and ability to recognize IPV, and (3) solutions they offer to victims of IPV.

## 2 Methods

The study used a cross‐sectional design in a survey format to collect information from dental professionals across Canada regarding IPV. To be eligible for the study, participants must have been a dentist, a dental hygienist, dental therapist, or dental assistants. Participants were excluded if they were not members of one of those four groups. From June 2025 to September 2025, dental professionals were surveyed across Canada using a validated and self‐administered survey on the SurveyMonkey platform. Participants were recruited using a non‐probability sampling technique and were approached by sharing the survey through provincial dental associations and through relevant Facebook groups.

The survey used was adopted and modified from a study completed by Alshouibi [16]. Additional questions were created based on the literature regarding IPV [14, 17]. The survey was further validated by having a group of dental students and professionals in a closely related field (e.g., forensic psychology, nursing, medicine, IPV researchers) complete the survey and provide comments and suggest modifications.

The final survey consisted of 54 questions and took about 10–15 minutes to complete. It included questions that examined demographics (e.g., age, gender, profession, years of dental experience, location of practice, practice setting), competency and willingness to identify IPV, and how professionals manage situations where they suspect IPV. The survey also included questions about previous training, education, and experiences with IPV.

Data were extracted from the platform and analyzed using descriptive statistics.

## 3 Results

A total of 327 dental professionals completed the survey. Table 1 depicts the demographics of the participants.

**TABLE 1:** Demographics of participants who completed the survey.

Measure	Item	Number	Percentage
Gender	Male	97	30
	Female	229	70
	Unidentified	1	0.31
Age	18–24	18	5
	25–34	70	21
	35–44	82	25
	45–54	64	20
	55–64	48	15
	65–75	33	10
	75+	11	3
	Rather not say	1	0.31
Profession	Dentist	183	56
	Dental hygienist	60	18
	Dental therapist	5	2
	Dental assistant	77	24
Years of experience	0–9	97	30
	10–19	77	24
	20–29	63	19
	30–39	43	13
	40+	46	14
Practice setting	Group private	174	53
	Solo private	120	37
	Public	4	1
	Other	29	8
Practice location	Urban	214	65
	Suburban	57	17
	Rural	51	16
	Other	5	2

Table 2 depicts the full set of answers for questions addressing knowledge and training regarding IPV. While a significant majority indicated they knew what IPV was (92%), only a minority indicated either having experienced (18%) or witnessed (36%) IPV at any point. A majority of participants reported no training about IPV in dental school (88%) and their career (81%). Of those who did receive training, it was primarily in the format of in‐class lectures during schooling (94%) or online courses during their career (51%).

**TABLE 2:** Responses to questions regarding knowledge and training.

Question	Response	Number	Percentage
What factors lead you to believe they were a victim of intimate partner violence?	Face and neck trauma (e.g., bruises, lacerations)	35	43
	Dental trauma (e.g., fractured teeth, oral mucosa lacerations)	32	40
	Behavioral indicators (e.g., scared, presence of a controlling spouse)	49	60
	Patient asked for help or described their situation	18	22
	Other	5	6
What do you consider to be the most important indicator that a patient is potentially a victim of intimate partner violence?	Face and neck trauma (e.g., bruises, lacerations)	64	23
	Dental trauma (e.g., fractured teeth, oral mucosa lacerations)	21	8
	Behavioral indicators (e.g., scared, presence of a controlling spouse)	146	52
	Patient asked for help or described their situation	41	15
	Other	8	3
I will notice signs that a patient is a victim of intimate partner violence	Strongly agree	14	5
	Somewhat agree	102	37
	Neither agree nor disagree	104	37
	Somewhat disagree	47	17
	Strongly disagree	12	4
What percentage of your patients do you believe are currently or have previously been victims of intimate partner violence?	0%–10%	158	54
	11%–20%	23	8
	21%–30%	26	10
	> 30%	13	4
	Unknown	71	24
In dental school, what IPV‐related training did you receive?	In‐class lecture	34	92
	Online course	5	14
	Community based presentations (e.g., representative from a shelter or police)	2	5
	Interprofessional education (e.g., working with medical students)	1	3
	Other	3	8
In your career, what IPV‐related training did you receive?	In person courses	20	35
	Online course	29	51
	Community based education (e.g., spokesperson from a shelter, police)	14	25
	Self‐education	24	42
	Other	2	4

Only a small number of respondents believed they had a patient who had experienced IPV (28%), while a large number believed they had never had a patient who was a victim of IPV (72%). Most respondents (57%) estimated < 10% of their patients are or have been patients of IPV, or used keywords such as “few,” “not many”, or “none”. Behavioral cues (e.g., presence of a controlling partner, looking scared) (60%) were the most common indicator or reason they believed that a patient was a victim of IPV. When asked about which identifying factor they believed to be the most important, a large majority (52%) of participants indicated that behavioral cues were the most important.

Dental professionals' responses regarding comfort and competence dealing with IPV victims are presented in Table 3. A significant majority indicated that they are either somewhat comfortable (32%) or uncomfortable (34%) directly asking patients about IPV‐related experiences. Similarly, a majority stated they were somewhat comfortable (34%) or uncomfortable (33%) initiating conversations related to IPV if they suspect it. When asked about their capacity to address IPV‐related concerns within their allotted time with each patient, there was a much more even spread of responses with a plurality somewhat agreeing (29%) that they had enough time. Furthermore, only a small number of respondents felt very confident (4%) or confident (11%) in their ability to detect IPV in practice. When asked about factors that limit their ability to detect IPV, there was an even spread of responses between fear of embarrassing or upsetting the patient, fear of putting the patient in danger, lack of education, and not knowing what to say. If a patient had disclosed they were a victim of IPV, a majority (43% strongly agree) of respondents indicated they would follow up with them in future appointments.

**TABLE 3:** Responses to questions regarding comfort and competence addressing IPV.

Question	Response	Number	Percentage
I have been trained on how to talk to patients about intimate partner violence	Yes	41	14
	No	247	86
To what extent do you feel comfortable asking patients if they are victims of intimate partner violence?	Very comfortable	13	5
	Somewhat comfortable	33	11
	Neither comfortable nor uncomfortable	93	32
	Somewhat uncomfortable	99	34
	Very uncomfortable	50	17
To what extent do you feel comfortable talking to patients about intimate partner violence if you suspect it?	Very comfortable	18	6
	Somewhat comfortable	47	16
	Neither comfortable nor uncomfortable	98	34
	Somewhat uncomfortable	94	33
	Very uncomfortable	31	11
I have enough time to address these concerns during my interactions with patients	Strongly agree	30	10
	Somewhat agree	84	29
	Neither agree nor disagree	66	23
	Somewhat disagree	73	25
	Strongly disagree	35	12
To what extent do you feel confident in your ability to recognize intimate partner violence?	Very confident	12	4
	Somewhat confident	31	11
	Neither confident nor unconfident	149	52
	Somewhat unconfident	75	26
	Very unconfident	21	7
What factors, if any, limit your confidence and ability to detect intimate partner violence?	Fear of making the patient uncomfortable or embarrassed	181	63
	Fear of putting the patient in danger	132	46
	Lack of education	143	50
	Not knowing what to say	149	52
	None	17	6
	Other	32	11
At what point, if any, do you conduct an explicit screening for intimate partner violence?	Initial visit	2	1
	Any visit	45	16
	When the patient presents with an injury	27	10
	Do not currently conduct screening	195	70
	Other	11	4
If a patient discloses intimate partner violence, I follow up with them in future appointments	Strongly agree	120	43
	Somewhat agree	71	25
	Neither agree nor disagree	69	25
	Somewhat disagree	8	3
	Strongly disagree	11	4
I offer discreet ways for patients to seek help in my practice	Yes	78	28
	No	196	72
If a partner is present with the victim, it limits my ability to speak to my patient about my concerns	Strongly agree	187	69
	Somewhat agree	59	22
	Neither agree nor disagree	19	7
	Somewhat disagree	4	1
	Strongly disagree	1	0.37
If a child is present with the victim, it limits my ability to speak to my patient about my concerns	Strongly Agree	102	38
	Somewhat agree	97	36
	Neither agree nor disagree	37	14
	Somewhat disagree	27	10
	Strongly disagree	7	3

A significant majority indicated that no screening (70%) is currently conducted within their practice. Almost three quarters of respondents indicated that they do not have procedures or ways for patients to discreetly seek help (72%). The presence of a partner (69% strongly agree) or child (38% strongly agree; 36% somewhat agree) was a barrier to addressing IPV‐related concerns; however, more professionals strongly agreed that the presence of a partner was more limiting (69% strongly agree).

Table 4 depicts the full set of responses regarding participants awareness of resources for IPV victims. When asked how they would respond to or help victims with IPV‐related concerns, participants often wrote that they would provide a list of resources, ask questions to better understand, help connect them to resources, and a slightly higher amount indicating they would express concern for their safety. Only 16% indicated that they were unsure about what they would do in this situation. When a patient disclosed that they were a victim of IPV, respondents equally chose that they would express concern for their safety, note it in the patient's chart, offer referrals and resources, and ensure the patient's safety when leaving.

**TABLE 4:** Responses to questions regarding awareness of resources.

Question	Response	Number	Percentage
If I suspect or am informed that a patient is experiencing intimate partner violence, I would respond by:	Expressing my concern for their safety	183	64
	Telling them to leave their partner	2	1
	Providing them with a list of resources/referral	136	47
	Helping connect them to a resource/referral	158	55
	Asking questions to gain a better understanding of the situation	149	52
	Unsure	46	16
	Other	124	
If a patient discloses intimate partner violence, I:	Do nothing	1	0.36
	Express concern for their safety	190	69
	Write it down in their chart	195	71
	Offer referral services	187	68
	Ensure they are safe when they leave	156	57
If I suspect intimate partner violence, I know what resources are available for victims	Strongly agree	27	10
	Somewhat agree	79	28
	Neither agree nor disagree	50	18
	Somewhat disagree	58	21
	Strongly disagree	66	24
If a patient needs helps, I know where to refer them	Strongly agree	31	11
	Somewhat agree	73	26
	Neither agree nor disagree	46	16
	Somewhat disagree	70	25
	Strongly disagree	60	21
Where would refer them?	Shelter	26	25
	Police	27	25
	Psychological support	4	4
	Online resources/numbers	29	27
	Clare's Law	1	1
	Other	19	18
I know the shelter near me where I can refer a patient, if needed	Strongly agree	46	17
	Somewhat agree	63	23
	Neither agree nor disagree	37	13
	Somewhat disagree	48	17
	Strongly disagree	83	30
If I suspect intimate partner violence, I help them create a safety plan	Strongly agree	23	9
	Somewhat agree	46	17
	Neither agree nor disagree	97	36
	Somewhat disagree	60	22
	Strongly disagree	44	16
I have an intimate partner violence protocol in my office	Strongly agree	8	3
	Somewhat agree	6	2
	Neither agree nor disagree	53	20
	Somewhat disagree	57	21
	Strongly disagree	146	54
I change my treatment approach if I know my patient is or has been a victim of intimate partner violence	Strongly agree	46	17
	Somewhat agree	77	29
	Neither agree nor disagree	91	34
	Somewhat disagree	26	10
	Strongly disagree	30	11
How do you change your approach?	Spend more time with the patient	70	56
	Use less isolation (e.g., no rubber dam)	6	5
	Offer Medications (e.g., anxiety medications, nitrous oxide)	6	5
	Offer breaks	21	17
	Other	21	17
I communicate differently with my patient if I know they are or have been a victim of intimate partner violence	Strongly agree	59	22
	Somewhat agree	80	30
	Neither agree nor disagree	86	32
	Somewhat disagree	21	8
	Strongly disagree	21	8

A slight majority somewhat agreed (28%) they knew the resources and referral sources available for victims of IPV; however, about half of participants either somewhat disagreed (24%) or strongly disagreed (21%). When asked where they would refer patients, more indicated they would refer them to shelters (25%), police (25%), and online resources (27%) and only a few indicated they would refer them to psychological services or police resources like Clare's Law. Participants who chose “other” (18%) often indicated they would do all of the above. When asked about their knowledge of local shelters, there was an even spread of responses between answers with a slight increase indicating they strongly disagreed they knew nearby shelters for referral (30%). In a follow‐up to “I offer discreet ways for patient to seek help,” participants were asked about what methods they offer, to which written responses often indicated that they offer referrals and resources, private conversations, and emergency services like the police.

Most participants (21% somewhat disagree; 54% strongly disagree) indicated that their place of work had no current protocol related to IPV. When asked about changing their treatment approach if they know the patient is a victim of DV, a majority indicated they would spend more time with the patients (56%), followed by offering breaks (17%), using less isolation (5%), and medications to calm patients (5%). When asked about changing their communication style, a plurality neither agreed nor disagreed (32%). When asked how they change their communication approach in an open‐ended question, it was often indicated they would take a slower, gentler, and more empathetic approach with thorough explanations.

**FIGURE 1:**
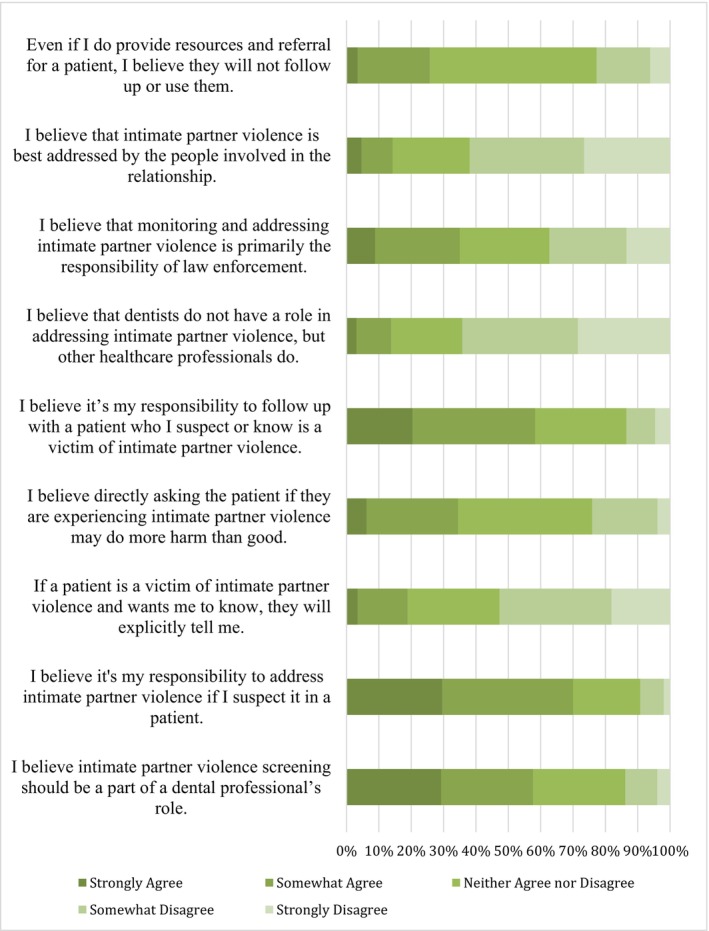
Responses regarding attitudes toward IPV.

Dental professionals' responses related to attitudes toward IPV are presented in Figure 1. Over half of the participants (61%) indicated they would like training regarding IPV. When asked about what methods they would like to see, continuing education courses or online modules were the preferred educational formats.

When asked if they felt that screening for IPV should be a part of their role, a plurality of respondents neither agreed nor disagreed (33%). However, of those who did indicate a direction, significantly more agreed (28%) or somewhat agreed (27%). A majority either strongly agreed (30%) or somewhat agreed (40%) that they felt it was their responsibility to address IPV‐related concerns if they suspected it in a patient. When asked if it was their responsibility to later follow up with a patient whom they suspected or knew was a victim of IPV, a slight majority of respondents indicated they somewhat agreed (38%). When asked if they believe that dental professionals do not have a role in IPV‐related concerns, but other healthcare professionals do, most selected somewhat disagree (36%) or strongly disagree (28%). Finally, answers were split as to whether participants felt IPV‐related concerns were best handled by law enforcement, with significant representation of each of somewhat agree (26%), neither agree nor disagree (28%), and somewhat disagree (24%).

A plurality somewhat disagreed (35%) that victims of IPV would explicitly disclose that to dental professionals. Additionally, a plurality of participants neither agreed nor disagreed (42%) that addressing or questioning IPV‐related concerns does more harm than good. When asked if IPV‐related concerns were best addressed by people involved in the relationship, most respondents somewhat disagreed (35%) or strongly disagreed (27%), or neither agreed nor disagreed (24%). Finally, a majority neither agreed nor disagreed that patients would not use the resources they provided to victims of IPV (52%).

## 4 Discussion

This study investigated dental professionals' knowledge and attitudes toward IPV in various practice settings. While other studies have examined dentists' preparedness to screen for IPV in practice settings, they have not addressed this topic from the viewpoints of other dental professionals or addressed solutions offered to victims [14, 16]. Therefore, the goals of this study were to examine dental professionals' knowledge regarding IPV, their competency and willingness to screen for IPV, and the solutions they offer to victims of IPV.

The first set of results relates to the prevalence of training for dental professionals. The main takeaway is that training is incredibly rare as most participants have not received any training related to IPV in their schooling or their career. For the small percentage who did, it was mainly in the form of lectures during schooling or courses for career‐based training. These results are consistent with previous research, which found low rates of training among dentists; however, the present study has built onto this by showing low rates of training not just among dentists, but also other dental professionals [14]. Extending the scope in this way demonstrates how there may be a need for a systemic change in how the dental profession understands and teaches about IPV. Low levels of IPV‐related education may hinder dental professionals from properly approaching and helping victims of IPV within their practice. One possible future direction of research would include examining how different academic institutions and career settings conduct training differently to help identify priority areas moving forward.

Secondly, there are low comfort levels around IPV‐related conversation and a lack of procedures related to IPV. When handling conversations related to IPV, most respondents did not feel confident or comfortable. Participants felt even more uncomfortable if there was a partner or child with the patient. When asked about other barriers to approaching these conversations, participants reported they worried they would embarrass the patient, put them in danger, did not know what to say or lacked education. This is consistent with previous research, which found that dentists were afraid to raise their concerns out of fear they would embarrass a patient or there was a child or partner present with the patient [15, 16]. This may hinder the success of training programs and education if dental professionals are not comfortable raising concerns or even reporting. These barriers and lack of comfort may explain the 70% of dental professionals who do not currently conduct screening and the 76% who do not have an IPV‐related protocol in their office. There does, however, appear to have been some increase in the proportion of dentists conducting screening over the past decades as prior research has found that around 87% of dentists do not screen for IPV [14, 15]. Nevertheless, these low screening levels indicate the need for more training and protocols to enhance dental professionals' comfort and guidance to screen and discuss IPV‐related concerns. At the same time, it may be incumbent upon dentists to take steps to familiarize themselves with possible ways to screen for IPV, and implement those within their own practices. Even if more training is not provided, it is important to at least attempt to create more attempts to screen and address IPV within practices.

Despite low comfort and training levels, participants generally seemed to be aware of resources available for IPV victims. However, when asked about specific local resources, there was a lack of awareness. This is an important gap because while a dental professional may know a shelter is a good resource, knowing where they are and how to refer patients is a crucial next step. Again, this may reflect low training levels and/or other barriers, such as time or attitudes. These findings indicate that, although further training may be the best outcome, it may still be important for dental professionals to research resources in their local communities, for example by reaching out to local groups aimed at supporting victims of IPV. Aside from training, the development of resource guides may also help dental professionals find and connect with local resources, such as shelters.

Generally, participants expressed positive attitudes toward IPV and victims of IPV. For example, 61% indicated they want more training related to IPV and expressed that they thought handling IPV‐related concerns was a part of a dental professional's role. This is important because the lack of screening aids and protocols may not be a result of resistance from professionals but rather training and education, and if further IPV‐related programs were available it seems as though many professionals would pursue them. It may also be worth conducting further research on how to optimize future training efforts. Dental professionals should take the opportunity to support victims of IPV and refer them to needed resources and support [10,18,19].

A limitation of the present study was that participants were selected via non‐probability sampling technique, which may be prone to self‐selection bias and may not accurately represent the entire population of dental professionals. The study also used a cross‐sectional design which has limits in establishing causality.

The present study indicates that dental professionals generally see themselves as having some role in identifying and responding to IPV and have a desire to be further trained on IPV. Despite these positive attitudes, dental professionals rarely receive proper training, and typically feel uncomfortable addressing IPV, and lack procedures and screening to properly handle cases involving IPV. Consequently, the development and provision of protocols and educational resources to dental professionals is strongly suggested. This highlights the need for accessible guides and education aimed at better informing dental professionals on IPV, in order to put them in a position to provide aid that is often needed and that they understand is important.

## Author Contributions

All authors made substantial contributions to the manuscript. This includes conceptualization, methodology, validation, investigation, resources, writing the original draft, reviewing, and editing. All authors have read and approved the final version of the manuscript.

## Funding

The study was partially supported by the University of Saskatchewan COD summer research program.

## Ethics Statement

The study was reviewed and approved by the Research Ethics Board (REB) at the University of Saskatchewan (Beh 5679).

## Conflicts of Interest

The authors declare no conflicts of interest.

## Data Availability

The data that support the findings of this study are available on reasonable request from the corresponding author. The data are not publicly available due to privacy or ethical restrictions.
